# Protein components of ribonucleoprotein granules from *Drosophila* germ cells oligomerize and show distinct spatial organization during germline development

**DOI:** 10.1038/s41598-019-55747-x

**Published:** 2019-12-16

**Authors:** Hieu D. L. Vo, Wahiduzzaman, Samuel J. Tindell, Jimiao Zheng, Ming Gao, Alexey L. Arkov

**Affiliations:** 1 Department of Biological Sciences, Murray State University, Murray, KY 42071 USA; 2 Biology Department, Indiana University Northwest, Gary, IN 46408 USA

**Keywords:** Germline development, Organelles

## Abstract

The assembly of large RNA-protein granules occurs in germ cells of many animals and these germ granules have provided a paradigm to study structure-functional aspects of similar structures in different cells. Germ granules in *Drosophila* oocyte’s posterior pole (polar granules) are composed of RNA, in the form of homotypic clusters, and proteins required for germline development. In the granules, Piwi protein Aubergine binds to a scaffold protein Tudor, which contains 11 Tudor domains. Using a super-resolution microscopy, we show that surprisingly, Aubergine and Tudor form distinct clusters within the same polar granules in early *Drosophila* embryos. These clusters partially overlap and, after germ cells form, they transition into spherical granules with the structural organization unexpected from these interacting proteins: Aubergine shell around the Tudor core. Consistent with the formation of distinct clusters, we show that Aubergine forms homo-oligomers and using all purified Tudor domains, we demonstrate that multiple domains, distributed along the entire Tudor structure, interact with Aubergine. Our data suggest that in polar granules, Aubergine and Tudor are assembled into distinct phases, partially mixed at their “interaction hubs”, and that association of distinct protein clusters may be an evolutionarily conserved mechanism for the assembly of germ granules.

## Introduction

Membraneless RNA-protein (ribonucleoprotein, RNP) structures are central to all aspects of cellular life and the assembly of different types of these structures has been the focus of intense investigation^1–5^. Different RNP assemblies show different degrees of structural homogeneity and dynamics ranging from stable uniform structures (such as ribosomes) to highly dynamic granules including processing bodies (P-bodies) and germ granules.

In particular, germ granules, which assemble in germ cells in different organisms and contain RNA and proteins required for germline development^6^, provide an important model to study the assembly mechanisms of large and dynamic RNPs. These granules themselves are quite diverse and range from the structures resembling liquid droplets to more structurally defined and less fluid structures or a combination of different phases in the same particle. The assembly mechanisms of germ granules have not been understood well. However, it has recently been shown that in *Drosophila* germ granules, formed at the egg’s posterior cytoplasm (germ plasm) referred to as polar granules, RNA molecules form homotypic clusters which contain multiple molecules of the same RNA species^7,8^. Further studies provided evidence that these homotypic RNA clusters are built up in the granules, starting from single RNA molecules, by a continuous seeding mechanism, which involves these RNAs’ 3’UTR elements responsible for RNA targeting to and clustering within the granules^9,10^. These studies provided important insights into RNA assembly in the polar granules. However, the assembly of protein components into the granules remains poorly understood.

Similarly to other types of germ granules, integral protein components of polar granules include Tudor (Tud)-domain containing polypeptide (Tud protein) and Piwi family protein Aubergine (Aub)^1,11^. Tud protein is a large molecular scaffold which consists of 11 Tud domains^12–15^. Each Tud domain is a 50–55 amino acid module, which is found in different proteins in many diverse organisms, and it has been shown to interact with methylated lysines or arginines of target proteins^16^. In particular, Tud protein interacts directly with symmetrically dimethylated arginine residues (sDMAs) of Aub^17–19^.

Here, using super-resolution confocal microscopy, we focused on the detailed distribution of protein polar granule components, Tud and Aub, in early embryos before and after germ cell formation stage. Since these proteins are direct interacting partners, we expected to see their complete overlap in polar granules. Surprisingly, in preblastoderm embryos before germ cells formation, we found that Tud and Aub form distinct clusters in the same granules which partially overlap. We further showed that after germ cells form, these clusters give rise to large cytoplasmic spherical granules with unexpected distribution of the two proteins: Aub shell (“donut/ring”-like in 2D sections) wrapping around the Tud core. Using size-exclusion chromatography and native-gel electrophoresis, we demonstrated that, irrespective of its methylation status, purified Aub forms homo-oligomers. This property of the Piwi family protein to oligomerize may contribute to the formation of this protein’s distinct clusters within the granules and to the assembly of the shell around the Tud scaffold in germ cells. To further characterize the mechanism of Aub-Tud interaction in the regions of the granules where these protein clusters associate, we generated all 11 Tud domains individually and tested their interactions with purified Aub. Our data demonstrated that six different Tud domains of Tud protein distributed along the entire Tud primary structure, associate with Aub indicating that one molecule of Tud may recruit multiple Aub homo-oligomers.

Our study shows that polar granule proteins can assemble in a more unique and non-homogeneous pattern within the granules than previously thought. Furthermore, this work suggests that some protein components of polar granules are engaged in the granule assembly by forming distinct phases mixed at certain regions (“interaction hubs”) and this may be a common strategy for the assembly of germ granules in different organisms.

## Results

### Tudor and Aubergine form distinct and partially overlapping clusters in polar granules of early *Drosophila* embryos

**Figure 1:**
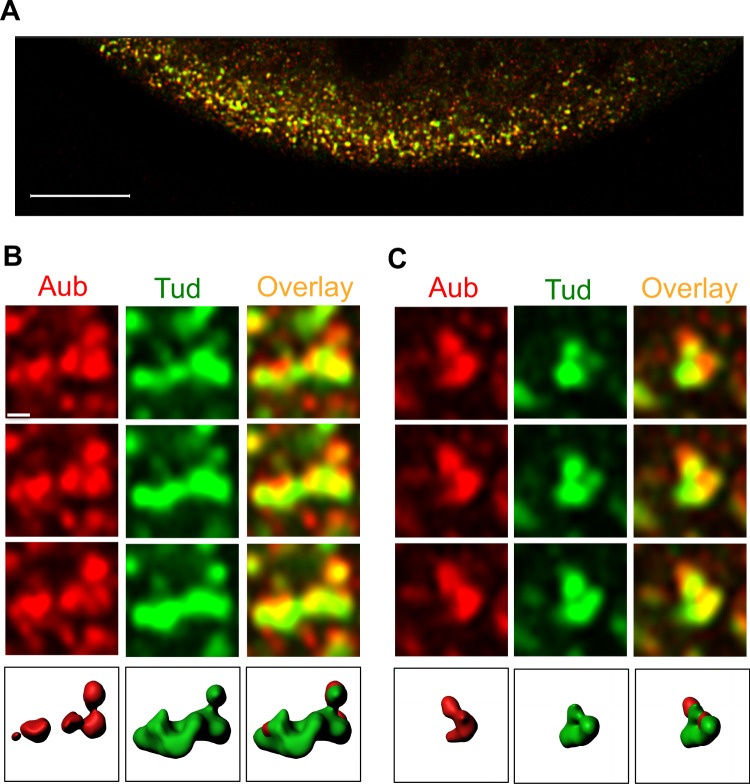
Integral protein components of polar granules, Tudor and Aubergine, form distinct partially overlapping clusters in the germ plasm of preblastoderm embryos. (**A**) Posterior germ plasm co-stained with anti-Aub antibody (red channel) and anti-GFP antibody to detect GFP-Tud (green channel). (**B**,**C**) Super-resolution confocal microscopy images of individual polar granules which show Aub and Tud clusters in the same granule (red and green channels respectively). Three consecutive optical sections, shown for each granule, demonstrate a partial overlap between Aub and Tud clusters. Bottom panels are 3D reconstructions of the granules with Imaris software (see also Supplementary Fig. S1). 7–11 sections per granule were used for 3D reconstruction. Scale bar in (**A**) is 10 µm. Scale bar in (**B**) is the same for all the panels in (**B**,**C**), including 3D reconstruction images, and is 0.4 µm.

To determine the detailed distribution of integral interacting polar granule proteins, Tud and Aub, we carried out super-resolution confocal imaging of the granules immunostained with anti-GFP antibody (to label functional GFP-tagged Tud that we previously described^20^) and specific anti-Aub antibody^21^. GFP-Tud shows the localization to germ plasm and germ cells^20,22^ as the untagged Tud^23^, indicating that GFP-Tud is a reliable marker of polar granules. In the preblastoderm embryos (before primordial germ cell formation), polar granules in the germ plasm at the embryo’s posterior unexpectedly showed distinct Tud and Aub clusters which only partially overlap (Fig. 1 and Supplementary Fig. S1). These distinct clusters and their partial overlap were clearly observed in multiple optical planes during the complete 3D reconstruction of different granules (7–11 optical sections per each granule were used to reconstruct 10 well-defined granules, Fig. 1 and Supplementary Fig. S1). Despite the limited overlap, Tud- and Aub-specific clusters assemble into the same polar granules, which vary in size and morphology at this early stage of embryo development, and their diverse 2D appearance and size agreed well with previously published electron microscopy (EM) section images at this stage^12,20,24^.

### Primordial germ cells show large spherical granules with Aubergine shell wrapping around Tudor core

**Figure 2:**
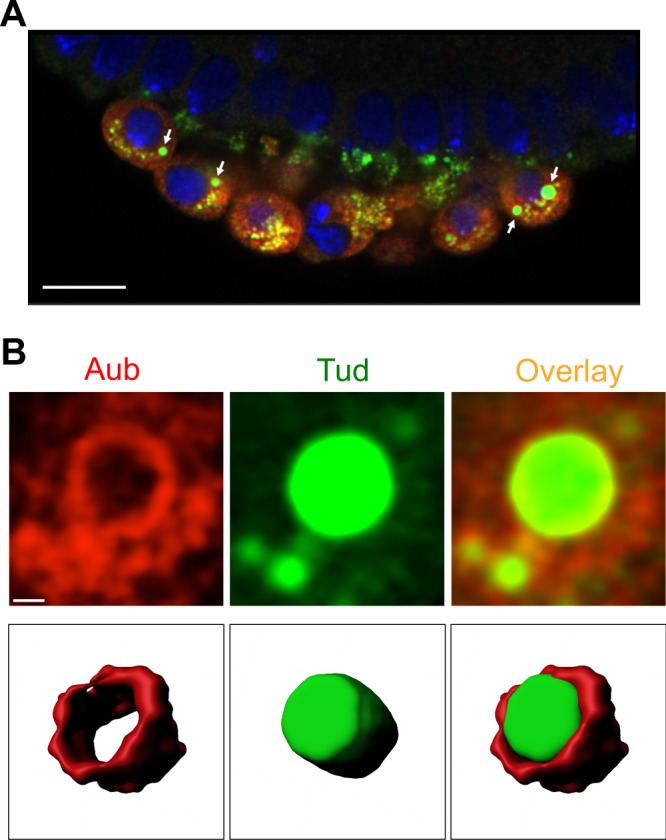
Germ cells in cellular blastoderm embryos show large cytoplasmic granules with Aubergine shell around Tudor core. (**A**) An optical section of primordial germ cells formed at the embryo’s posterior co-stained with antibodies against Aub (red channel) and GFP-Tud (green channel). DAPI stains the nuclei of germ cells and somatic cells at the cortex of the embryo (blue channel). Large granules assembled in the cytoplasm of germ cells are indicated with arrows. (**B**) Top panels are optical sections obtained with super-resolution confocal microscopy which show Aub shell (red channel) wrapping around Tud core (green channel). Bottom panels are tilted 3D reconstructions of Aub shell (red), Tud core (green) and the composite granule internal segments to show the internal shell-core architecture of the granule imaged at the top panels. 10 internal optical sections were used for 3D reconstructions. Scale bar in (**A**) is 10 µm and in (**B**) it is 0.5 µm (see also Supplementary Fig. S2).

At the cellular blastoderm stage after germ cell formation, the Tud/Aub-containing germ granules were exclusively cytoplasmic, consistent with previous observations^22,25^, and in addition to multiple germ plasm-like granules, germ cells exhibited new large spherical or nearly spherical granules that consist of Tud core surrounded by Aub shell located on the surface of the Tud core (there are about 1–2 of these large granules per germ cell, Fig. 2A). These granules’ diameter in equatorial sections is on average 1.13 μm±0.07 (s.e.m.), n = 16, with the granules’ diameter ranging from 0.79 μm to 1.71 μm (Fig. 2 and Supplementary Fig. S2). The diameter of these granules agrees well with previously described EM images of the large cytoplasmic spherical germ granules at this developmental stage^26^. Interestingly, in these granules, EM sections obtained several decades ago, show electron-dense rim and electron-lucid core, which, as our data now suggest, include Aub- and Tud-specific regions respectively. While these regions are distinct, Aub shell appears to interact with Tud core at the Aub/Tud border (Fig. 2 and Supplementary Fig. S2).

### Aubergine forms homo-oligomers

**Figure 3:**
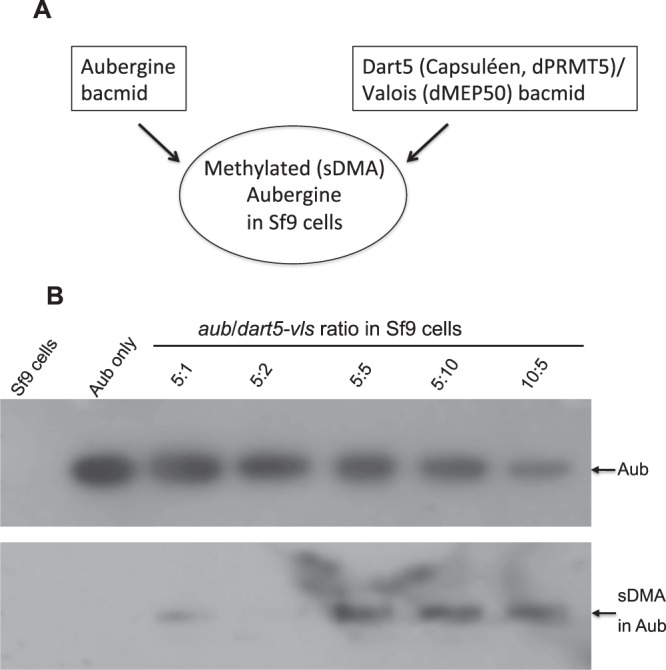
Generation of Aubergine with symmetrically dimethylated arginines in Sf9 cells. (**A**) Aubergine was co-expressed with sDMA-generating methyltransferase Dart5 and methylosome protein Valois in Sf9 cells. (**B**) Expression of *aub/dart5-vls* bacmids using different multiplicity of infection (MOI) ratios resulted in the generation of sDMAs in Aub purified from Sf9 cells reaching the saturation levels at the MOI ratio of 5:5 for both bacmids. sDMAs in Aub were detected by Western blot with SYM11 antibody (bottom panel). Aub was detected with anti‐c‐Myc antibody (top panel). Negative controls (just Sf9 cells extracts and Aub from Sf9 cells expressing *aub* bacmid without *dart5-vls* bacmid) are shown in the first two lanes.

An important mechanism that may contribute to the formation of protein-specific clusters in the granule and the assembly of polar granules, shown in Figs. 1 and 2, is the protein multimerization or oligomerization. In particular, protein oligomerization was found to play a crucial role during the assembly of another membraneless organelle, nucleolus^27,28^. Although purified full-length Tud is a monomeric protein under native conditions^24^, it contains 11 Tud domains, presumably providing a multivalent scaffold to interact with different granule components. On the other hand, purification of full-length Aub for a biochemical analysis has been challenging^29^ and, therefore, oligomerization properties of Aub have not been tested. In addition, the biochemical analysis of Aub is further complicated by the need to introduce important symmetrically dimethylated arginines (sDMAs) in Aub that are required for its direct interaction with Tud. To obtain sufficient quantities of full-length Aub suitable for biochemical analysis, we chose baculovirus expression system and insect Sf9 cells and were able to express and purify Aub^20^. However, Sf9 cells lack methyltransferase activity required for the generation of sDMAs^30,31^. Therefore, to produce sDMA-containing Aub, together with Aub bacmid, we co-expressed *Drosophila* PRMT5 ortholog Dart5/Capsuléen (Csul), shown *in vivo* to be required for the generation of sDMAs in Aub^31^, and Valois (Vls), *Drosophila* MEP50 ortholog and required Dart5/Csul partner protein^32–35^. This strategy successfully resulted in the production of sDMAs in Aub detected with anti-sDMA SYM11 antibody (Fig. 3).

**Figure 4:**
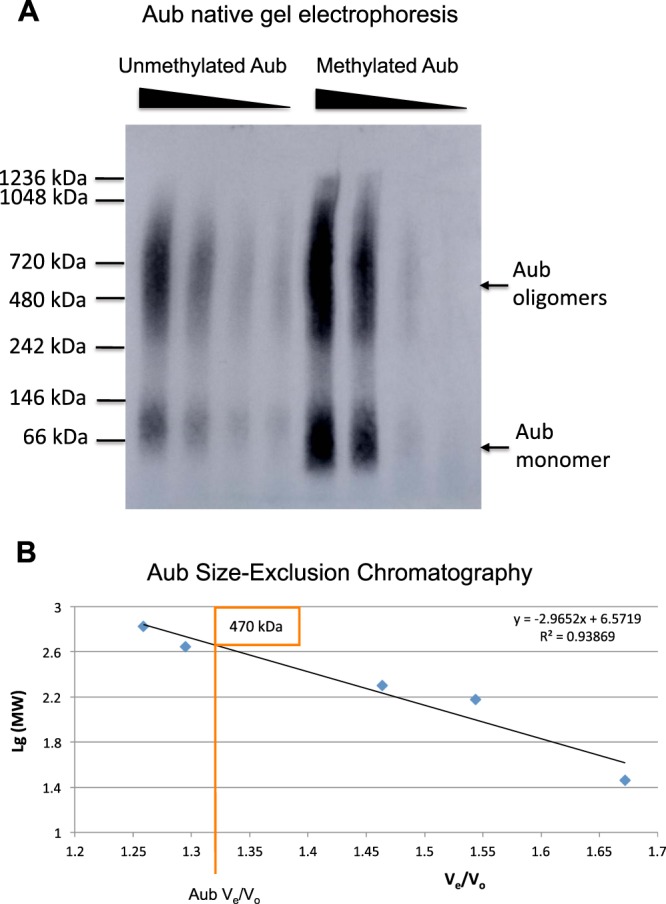
Aubergine forms homo-oligomers irrespective of its methylation status. (**A**) Western-blot detection of Aub homo-oligomers resolved with BN-PAGE. Unmethylated and sDMA-Aub (methylated) can form homo-oligomers under native conditions. Aub efficiently oligomerizes at different concentrations in samples shown in different lanes for both unmethylated and methylated Aub. (**B**) Native size-exclusion chromatography of sDMA-Aub confirms its homo-oligomerization properties. Logarithms of the molecular masses of standard proteins and their corresponding ratios of elution volumes (V_e_) and void volume (V_o_), measured with the elution volume of blue dextran, were plotted from size-exclusion chromatography experiments (indicated with blue squares). V_e_/V_o_ value of the Aub peak’s central fraction corresponded to the homo-oligomer’s molecular mass of ~500 kDa.

Next we asked whether purified methylated Aub (monomeric Aub is ~100 kDa) can form homo-oligomers. Using blue native polyacrylamide gel electrophoresis (BN-PAGE), we demonstrated that Aub forms homo-oligomers which migrate within the range of 500–700 kDa complexes (Fig. 4A). Furthermore, In order to confirm the oligomerization properties of Aub detected with BN-PAGE, we carried out size-exclusion chromatography (SEC) of purified methylated Aub under native conditions (Fig. 4B). The SEC experiments were in good agreement with the BN-PAGE data and showed that Aub is eluted as a large homo-oligomeric complex over several fractions with the central peak fraction corresponding to ~500 kDa (Fig. 4B).

### Methylation of aubergine is not required for its oligomerization

We further tested if sDMA modifications in Aub are required for Aub homo-oligomerization. To this end, we isolated unmethylated Aub from Sf9 cells lacking *dart5/vls* bacmid (Fig. 3). Subsequently, we showed that unmethylated Aub was able to form homo-oligomers which were indistinguishable from those formed by methylated Aub (Fig. 4A). Interestingly, both methylated and unmethylated Aub oligomerizes efficiently within the broad range of concentrations (Fig. 4A) indicating that the formation of 500–700 kDa Aub oligomers is not concentration-dependent.

### Aubergine interacts with multiple tudor domains distributed along the entire length of Tudor

**Figure 5:**
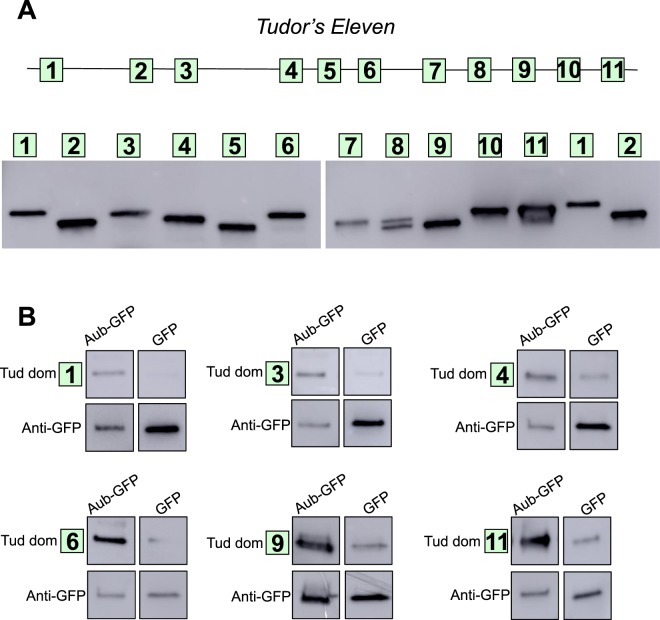
Aubergine interacts with multiple Tudor domains distributed along the entire Tudor primary structure. (**A**) Top: a diagram of Tud protein with the location of all 11 Tud domains indicated as squares. Bottom: each Tud domain was individually expressed and partially purified for binding experiments with Aub. All Tud domains had a His tag at the N-terminus and were detected with anti-His tag antibody in a western-blot experiment. Right panel shows Tud domains 7–11 and domains 1 and 2, also shown on the left panel, for direct comparison of all Tud domains used for Aub binding experiments since two gels were used to accommodate all the domains. (**B**) Tud domains 1, 3, 4, 6, 9 and 11 bind to Aub *in vitro*. All Tud domains were added to anti-GFP beads associated with functional GFP-Aub purified from fly ovaries^24,25^ and bound domains were detected by western blot with anti-His tag antibody. Maternally-expressed GFP, purified from the ovaries in parallel with GFP-Aub, and bound to the anti-GFP beads was used as a negative control for the binding experiments. GFP proteins were detected with anti-GFP antibody. Each binding experiment is demonstrated as a group of four western-blot segments from the same gel showing a given Tud domain or GFP-Aub or just GFP. Corresponding full-length blots are shown in Supplementary Figs. S3 and S4. Full-length blots corresponding to binding experiments for all the other Tud domains that failed to bind to Aub are shown in Supplementary Figs. S5 and S6.

Our super-resolution microscopy experiments showed that Aub clusters partially overlap with Tud clusters within the same polar granules (Fig. 1 and Supplementary Fig. S1). In addition, in primordial germ cells, Aub shell apparently associates with Tud core in the large cytoplasmic granules (Fig. 2 and Supplementary Fig. S2). Consistent with these data, Aub was shown to interact with Tud directly^17–19^, however, detailed molecular analysis of this interaction has been limited to the C-terminal fragment of Tud protein (Tud domains 7–11) and methylated Aub peptide. To comprehensively analyze the mode of interaction of full-length Aub and Tud, we expressed and purify all 11 Tud domains of Tud protein (Fig. 5A) and determined whether full-length Aub binds to them *in vitro*. Independent binding experiments (2–4 experiments for each Tud domain) demonstrated that Aub specifically binds to Tud domains 1, 3, 4, 6, 9 and 11 (Fig. 5B and Supplementary Figs. S3 and S4) supporting our previous conclusion based on the analysis of large Tud fragments-Aub binding experiments, that Tud segment, which includes Tud domains 2–6, is involved in interaction with Aub^21^. Here we show that multiple Tud domains along the entire primary structure of Tud are engaged in the interaction with Aub.

## Discussion

This work focuses on the high-resolution imaging and *in vitro* analysis of the assembly of interacting proteins, Piwi family protein Aub and scaffold protein Tud, in the RNA-protein granules (polar granules) in the germline. Aub and Tud are the principal components of the granules and since they interact, it was expected that they would be homogenously distributed within the granules. Surprisingly, we found that, while in the same granules and partially overlapping, these proteins form distinct mutually exclusive clusters in the germ plasm before germ cell formation. In addition, after germ cells form, Aub and Tud show even more striking segregation pattern within the large sphere-like granules that form in the cytoplasm of germ cells. In these granules, Aub is at the surface of the sphere, wrapping around a large Tud core.

Classic EM images of the germ granules in *Drosophila* germ cells from Mahowald’s lab demonstrated large spherical cytoplasmic organelles, which in EM sections, appeared as donut- or ring-like structures with electron-dense rim around an electron-lucid core^26^. These granules had the diameter of 0.75 μm – 1 μm in EM sections and are in good agreement with the Aub-Tud large granules described here (1.13 μm on average), suggesting that one of the components of the electron-dense rim is Aub. Furthermore, consistent with our data, similar donut-like Aub granules were described in germ cells using confocal microscopy^25,36^. Although these large spherical granules are quite prominent, they are not as abundant as much more numerous germ plasm-like small polar granules in the cytoplasm of germ cells, therefore, the test of their functional significance awaits further investigation.

Interestingly, other proteins can assemble at the surface of large granules, that are different from polar granules, with ring-like distribution visualized in optical sections which resemble electron-dense rim of the corresponding granules imaged with EM. In particular, in *Drosophila melanogaster*, while Tud and Aub are exclusively cytoplasmic, germ granule proteins Oskar (Osk) and Vasa (Vas) can form granules in the nuclei of germ cells, which are referred to as nuclear bodies^22,25,36^. These nuclear bodies are similar in EM sections to cytoplasmic large polar granules described above^26^, and also show an electron-dense rim with Osk and Vas ring-like distribution with the characteristic donut-like morphology in optical sections. Furthermore, in *C. elegans* germ granules (P granules), using high-resolution lattice light sheet microscopy, MEG-3 protein was shown to be assembled at the surface of the PGL-3 protein core and these proteins only partially overlap within the P granules^37^. MEG-3/PGL-3 distribution in P granules resembles Aub/Tud assembly in the large cytoplasmic granules in *Drosophila* germ cells and suggests conservation in the molecular mechanisms of protein assembly in the large germ granules.

Interestingly, other “non-germ” RNP granules, including mammalian P-bodies, stress granules and nucleoli show heterogeneity in their protein distribution within the granules^4,27^. In particular, there is evidence that *Xenopus* nucleoli consist of different immiscible liquid-like phases that form core-shell arrangement, with Nucleophosmin NPM1 protein phase (shell) enveloping the FIB1 protein clusters (core). What may be responsible for the shell/core arrangement of the germ and nucleolus RNP granules? Analysis of the nucleolus protein phases provided evidence that their different hydrophobicity and surface tensions result in their distinct incorporation into the nucleolus structure which can be mimicked using different types of oil mixed with water^27^. However, the FIB1 clusters can also “age” and transition to solid-like state over time. Another mechanism to form distinct compartments within the RNP granules is based on the reentrant phase transition of RNPs which can be controlled by RNA^38^. In this case, titration of an RNA-binding protein with increasing RNA concentration can initially result in the formation of a positively charged RNP droplet (due to a positively charged RNA-binding protein amino acid residues) which subsequently, at higher RNA concentration, leads to the change of the RNP charge from positive to negative. This charge inversion of the RNP granule causes the eventual dissolution of the granule. However, during this process, internal compartments form inside the RNP granules and at certain RNA concentrations these granules can exist for more than two hours^38^, which can be sufficiently long for the time scale of many cellular and developmental processes. The physical principles described above, based on different liquid phases’ surface tensions in aqueous environment and the reentrant phase transition, were used to describe behavior of spherical granules^27,38^ and may be contributing to distinct cluster and core-shell architecture of Aub-Tud RNP polar granules reported here. In fact, Tud scaffold core may stabilize Aub shell to prevent that from dissolution. However, some important aspects of Tud and Aub distribution as distinct clusters in polar granules before germ cell formation, when these proteins are not embedded into spherical granules but rather overlap in “interaction hubs” forming amorphous diverse granules (Fig. 1 and Supplementary Fig. S1), await further biochemical and biophysical analysis.

Our data show that Aub forms homo-oligomers under native conditions. It remains to be determined whether this oligomerization property of Aub is required for the assembly of polar granules. Interestingly, similarly to Aub homo-oligomerization, nucleolar shell NPM1 protein forms pentameric homo-oligomers^39^ and this oligomerization is required for NPM1 to form the protein liquid droplets *in vitro* and to efficiently localize to the nucleolus^27,28^.

**Figure 6:**
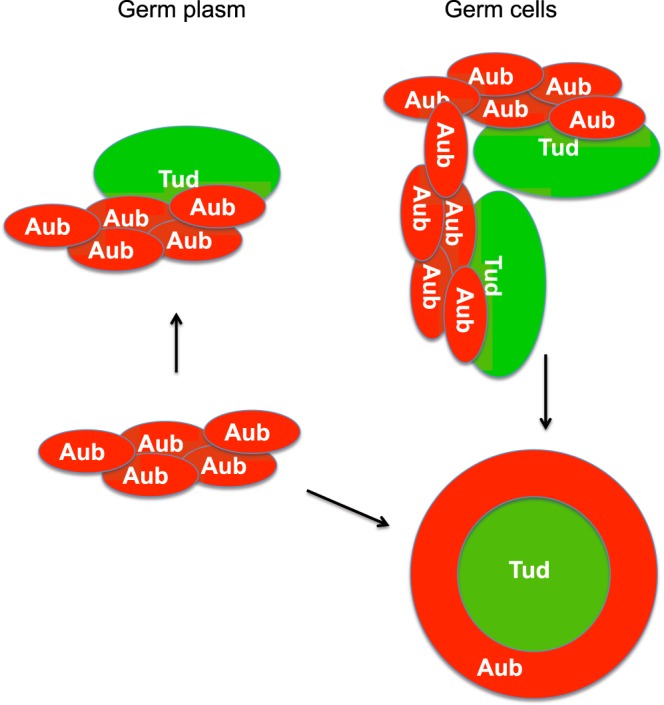
A model for the assembly of Aubergine and Tudor clusters into polar granules in germ plasm and germ cells. In germ plasm, Aub homo-oligomers and scaffold Tud assemble into distinct RNA-protein clusters which have transitioned from liquid partially mixed phases into gel- or solid-like structures fused at the “interaction hubs”. These “interaction hubs” involve multiple Tud domains interacting with Aub homo-oligomers. Further concentration of Aub/Tud clusters in the cytoplasm of germ cells by dynein-driven active transport^41^ results in large spherical or nearly spherical granules with Aub shell-Tud core architecture. In this granule, different Aub homo-oligomers may associate with each other and recruit free homo-oligomers from the cytoplasm forming a cage around Tud core and contributing to the stability of the shell. Also, Tud core partially associates with Aub shell further stabilizing the structure of the granule.

We also demonstrate that Aub homo-oligomers are insensitive to Aub methylation status and form when Aub contains sDMAs required for interaction with Tud domains of Tud protein. This may indicate that in polar granules, Tud binds to methylated Aub oligomers rather than to monomeric Aub (Fig. 6). Furthermore, here we show that six Tud domains (1, 3, 4, 6, 9 and 11) of Tud can bind to Aub. Consistent with previous methylated Aub peptide-Tud domains 7–11 binding studies^19,40^, we did not detect interaction of domain 10 to full-length Aub presumably due to this domain’s incomplete sDMA-binding pocket^40^ and show Aub interaction with domains 9 and 11. Although we could not detect binding of Aub to the other five Tud domains of Tud protein (Supplementary Figs. S5 and S6), it was previously shown that methylated Aub peptides can bind to Tud domains 7 and 8^40^. Therefore, based on our and previous data, we propose that one molecule of Tud protein contains at least eight Tud domains that may potentially bind to multiple Aub homo-oligomers. However, future research will be required to determine the precise stoichiometry of Tud/Aub complexes in polar granules.

The formation of distinct Tud and Aub clusters in the same polar granules is intriguing and was unexpected since these proteins are *bona fide* interacting granule proteins. Tud and Aub may undergo phase transitions during the assembly of polar granules, forming, together with their interacting ligands, two distinct phases. Our data are consistent with the model that while these Tud and Aub phases are immiscible with the surrounding cytoplasm, they may be partially mixing and wetting each other, thereby partially overlapping in the “interaction hubs” (Fig. 6). Non-spherical shape of Aub- and Tud-labeled polar granules in the germ plasm before germ cells formation may indicate that Aub and Tud phases, at least to some degree, have transitioned to gel-like or solid-like state. In support of this model, it was observed that polar granules in the germ plasm of early embryos are more structured than liquid droplets and seem to contain both liquid and hydrogel-like regions^22^.

After germ cells form in the embryo’s posterior, the formation of large granules with Aub shell and Tud core may be driven by the concentration-dependent assembly since germ cells actively accumulate polar granule components using dynein-dependent transport^41^. During this process Aub homo-oligomers (which themselves form even at low concentration) may be interacting with each other to stabilize the shell of the granule and free Aub oligomers may be recruited to assemble around Tud scaffold (Fig. 6).

Previous imaging analysis of the polar granules demonstrated that RNA granule components form homotypic clusters in the granules^7,8^. Here we show that interacting protein components of polar granules form distinct clusters within the same granules which can be subsequently distributed into the large shell-core architecture during development. Future studies will establish how the RNA and protein clusters together determine the granule morphology and how this clustering contributes to the function of polar granules in germ cell development.

## Methods

### Microscopy

Microscopy was carried out with Zeiss LSM 880/super-resolution Airyscan module system with inverted laser scanning confocal microscope AxioObserver and Plan-Apochromat 63 ×/1.4 Oil DIC M27 objective at Zeiss Microscopy Customer Center (Thornwood, NY). Images were analyzed and 3D reconstruction was carried out using Imaris software (version 9.3.1, Oxford Instruments) and HP Z8 workstation. For a 3D reconstruction, 7–11 optical sections per a well-defined granule were used using Surfaces option in Imaris. For each granule, Aub and Tud clusters were reconstructed separately and subsequently composite images of the complete granules were generated. For microscopy, preblastoderm and cellular blastoderm embryos expressing functional full-length GFP-Tud^20^ were immunostained with rabbit anti-GFP antibody (ab290) from Abcam (1:5000) and rat anti-Aub antibody (1:1000)^21^. This whole-mount immunostaining methodology was described previously^42^.

### Expression and purification of methylated Aubergine

Expression of Aub using baculovirus expression system was described and the Aub construct had N-terminal His‐, Myc‐, and 3xFLAG‐tags^20^. For protein expression, either Sf9 cells (Thermo Fisher Scientific) or TriEx^TM^ Sf9 cells (Novagen, Millipore Sigma) were used. To generate sDMAs in Aub, Aub bacmid was co-expressed with a bacmid that was constructed using pFastBac™ Dual vector (Thermo Fisher Scientific) and *Drosophila dart5(csul)* and *vls* cDNAs which allow expression of Dart5(Csul) methyltransferase and methylosome protein Vls from the same bacmid in Sf9 cells. When the virus titer was above 10^8^ pfu/ml, *aub* and *dart5*(*csul)-vls* viral stocks were mixed to transfect Sf9 cells at the multiplicity of infection (MOI) *aub*/*dart5*(*csul)-vls* ratios of 5:1, 5:2, 5:5, 5:10, 5:20, and 10:5. Based on the results of this analysis, to produce methylated Aub, the *aub*/*dart5*(*csul)-vls* MOI ratio of 5:7.5 was routinely used. For western-blot detection of sDMAs in Aub, anti-sDMA antibody SYM11 (Millipore, 1:1000) was used.

### Buffers used for aub purification

**Table:** 

Nuclease solution	0.1 M MgCl_2_, 5U universal nuclease (Thermo Fisher Scientific) in PBS
IP wash buffer	0.1% Tween-20, 1% IGEPAL CA-630, 0.1 M Glycine, 1 M NaCl in PBS
ATP buffer	5 mM ATP, 15 mM MgCl_2_, protease inhibitors, 1 mM phenylmethanesulfonyl fluoride (PMSF) in IP Wash Buffer
c-Myc peptide elution buffer	0.1 mg/ml c-Myc peptide, 300 mM Imidazole in PBS
Aub Binding Buffer (ABB)	1 M NaCl, 50 mM NaH_2_PO_4_, 10 mM Imidazole, pH 8
Arginine Wash Buffer (AWB)	500 mM NaCl, 50 mM NaH_2_PO_4_, 40 mM Imidazole, 250 mM Arginine, pH 8
Non-Arginine Wash Buffer (NonWB)	500 mM NaCl, 50 mM NaH_2_PO_4_, 40 mM Imidazole, pH 8
Aub Elution Buffer (AEB)	500 mM NaCl, 50 mM NaH_2_PO_4_, 300 mM Imidazole, pH 8
Tris-buffered saline (TBS)	25 mM Tris/Tris-HCl, 0.13 M NaCl, 2.7 mM KCl

### Aub purification

Aub protein was purified using anti-c-Myc immunoaffinity (MBL) and Ni-NTA purification systems (Thermo Fisher Scientific). Cells expressing methylated Aub (~1.5 ml) were homogenized in 6 ml of lysis buffer (IP wash buffer with protease inhibitors) using a sonicator. The cell extract was then incubated with 70 µl nuclease solution at 4 °C for 30 min with rotation. Cell debris was then separated by centrifugation at 16100 g at 4 °C for 12 min. The supernatant was then pipetted out and the debris was extracted again by sonication in 2 ml of lysis buffer. The extract was centrifuged and the supernatant fractions were combined. The combined supernatant was then filtered using 0.45 µm Millex filter unit.

Next, a gravity column with anti-c-Myc agarose was prepared according to the manufacturer’s protocol guidelines (MBL). The filtered lysate was slowly added to the column containing anti-c-Myc antibody beads. The flow-through fraction was then applied onto the column to increase the amount of Aub bound to the resin. The beads were washed twice with 3 ml IP wash buffer followed by the incubation with the ATP buffer to remove heat shock proteins. After the ATP buffer was added, the column was incubated at 27 °C for 15 min. Subsequently, the column was washed three times with 3 ml IP wash buffer per each wash. Aub was then eluted with 2 ml c-Myc peptide elution buffer and the elution fraction was concentrated using an Amicon Centrifugal Filter (10,000 Da Molecular Weight Cutoff, Millipore). During filtering, the final concentration of imidazole in the eluate was brought to less than 5 mM with PBS and the protein solution was supplemented with 400 mM NaCl, 40 mM NaH_2_PO_4_, 5 mM 2-mercaptoethanol and protease inhibitors including 1 mM PMSF.

Another gravity column was used for His-tag protein purification procedure as the next step. The column was prepared with 800 µl Ni-NTA beads as previously described^24^ except the column was equilibrated in ABB buffer. The c-Myc elution mixture was then slowly added to the beads and allowed to bind at 4 °C for 45 min with rotation. The beads were then drained and washed with 3 ml AWB followed by 5 ml NonWB. Aub was then eluted with 1.3 ml AEB and was concentrated using an Amicon Centrifugal Filter (10,000 Da Molecular Weight Cutoff). During filtering and protein concentration, the final concentration of imidazole in the eluate was diluted to less than 5 mM with TBS which was used as a storage buffer for final purified Aub sample.

### Blue native polyacrylamide gel electrophoresis (BN-PAGE) and size-exclusion chromatography of Aubergine

BN-PAGE methodology^43^ was used to analyze oligomerization properties of Aub purified with anti-c-Myc immunoaffinity protocol described above. Aub samples were loaded onto NativePAGE^TM^ 3–12% Bis-Tris protein gels (Thermo Fisher Scientific) and electrophoresis was carried out according to the manufacturer’s instructions followed by western-blot experiment to detect Aub with anti-Flag antibody (Sigma, 1:10000).

For native SEC, Superose^TM^ 6 Increase 3.2/300 size-exclusion column (GE Healthcare) was used. 50 µl of Aub, purified with immunoaffinity purification protocol followed by additional Ni-NTA purification as described above, was loaded onto the column and the chromatography was carried out with 40 µl/min flow rate at 4 °C in TBS. In addition, using the conditions specified above for Aub SEC, 5 size standards including thyroglobulin (669 kDa), apoferritin (443 kDa), β-amylase (200 kDa), alcohol dehydrogenase (150 kDa) and carbonic anhydrase (29 kDa) were used to calibrate the column similarly to previously described methodology^24^. Blue dextran’s (2,000 kDa) peak elution volume was used as void volume (V_o_) to calculate elution volume (V_e_)/V_o_ ratios for the standards and purified Aub. Aub peak fractions were determined by western-blot experiment using anti-Flag antibody (1:10000).

### Expression and partial purification of tudor domains

Tud domains 1–11 were cloned separately into the pET SUMO vector using the TA cloning method (Thermo Fisher Scientific) as follows. From *tud* cDNA encoding full-length Tud protein (2515 amino acids), different Tud domains constructs were generated to encode the following specific domains. Domain 1 (amino acids 1–217), domain 2 (amino acids 405–585), domain 3 (amino acids 592–772), domain 4 (amino acids 1002–1192), domain 5 (amino acids 1153–1328), domain 6 (amino acids 1299–1484), domain 7 (amino acids 1605–1785), domain 8 (amino acids 1791–1964), domain 9 (amino acids 1976–2160), domain 10 (amino acids 2140–2340), and domain 11 (amino acids 2335–2515). For expression, Champion^TM^ pET SUMO expression system (Thermo Fisher Scientific) was used and all domains had N-terminal His- and SUMO-tags.

All Tudor domains were expressed and partially purified with slight modifications of the earlier method^44,45^. The plasmids with domain constructs were transformed into *E. coli* BL21 (DE3) competent cells. Transformed BL21 (DE3) cells were cultured overnight at 37 °C with vigorous shaking at 220 rpm in LB broth medium containing 50 μg/ml kanamycin. Secondary cultures of these cells were grown by adding 1% of primary cultures and 50 μg/ml kanamycin at 37 °C with shaking at 220 rpm. When their absorbance reached 0.6, 0.25 mM isopropyl-1-thio-β-D-galactopyranoside (IPTG) was added to induce expression of recombinant proteins at 37 °C for 4 hr. Those cultures were centrifuged at 8000 g at 4 °C for 15 min. Pellets were resuspended in lysis buffer (50 mM Tris/Tris-HCl, 500 mM NaCl, 5% glycerol, 0.1 mg/ml lysozyme, 1% of Triton X-100, pH 7.4), and sonication was carried out on ice using ultrasonicator for 10 min (30 sec on, 30 sec off). After sonication and centrifugation at 4 °C for 20 min, pellets were discarded and supernatant was collected for further purification as follows.

The filtered supernatant samples were loaded onto Ni-NTA column pre-equilibrated with the column buffer (50 mM Tris/Tris–HCl, 500 mM NaCl, 5% glycerol, pH 7.5). For all domains, the unbound protein was washed with 10 mM imidazole solution. Domains 1 and 2 were eluted with 100 mM imidazole whereas other domains were eluted with 50 mM imidazole.

### Aub-tudor domains binding experiments

Purification of functional and methylated GFP-Aub^24,25^ and just GFP (negative control) from fly ovaries with anti-GFP beads (MBL) and the subsequent binding assays were carried out essentially as described for GFP-tagged proteins^24^. In particular, ~300 µl ovaries expressing either GFP-Aub or just GFP were homogenized in 800 µl lysis buffer (IP wash buffer, protease inhibitors including 1 mM PMSF) and cellular debris was then separated by centrifugation at 16100 g at 4 °C for 10 minutes. The supernatant was then pipetted out and the debris was extracted once again. Subsequently, the extract solution was filtered using 0.45 µm Millex filter unit.

60 µl anti-GFP agarose beads were then added to the filtered lysate and the mixture was incubated at 4 °C for 45 min. After incubation, the beads were washed three times with 400 µl IP wash buffer followed by three additional washes with 400 µl binding buffer (PBS + 0.05% IGEPAL CA-630). After the final wash, equal amounts of anti-GFP beads conjugated to a given GFP protein were used for 50 µl binding reactions with purified Tud domains. Binding reactions were carried out at 4 °C for 30 min with gentle disturbance every 10 min followed by 4 washes with binding buffer (400 µl for each wash). Bound Tud domains and GFP proteins were subsequently eluted with 30 µl SDS sample buffer for western-blot analysis. During western-blot experiments, Tud domains and GFP proteins were detected with anti-His-tag antibody (MBL, 1:7500) and anti-GFP antibody (Abcam, 1:50000) respectively.

## Supplementary information

Supplementary Information

## Data Availability

Materials and other data will be made available to readers upon request, which should be addressed to Alexey L. Arkov (email: aarkov@murraystate.edu).
