# Supplementary material for: Protein components of ribonucleoprotein granules from Drosophila germ cells oligomerize and show distinct spatial organization during germline development
Source: Sci Rep. 2019 Dec 16;9:19190. doi: 10.1038/s41598-019-55747-x (PMC6915754; doi:10.1038/s41598-019-55747-x)
Supplement: Supplementary file 1 — Supplementary Information [file 41598_2019_55747_MOESM1_ESM.pdf]

Supplementary Information

Protein components of ribonucleoprotein granules from *Drosophila* germ cells oligomerize and show distinct spatial organization during germline development

Hieu D. L. Vo[1], Wahiduzzaman[1], Samuel J. Tindell[1], Jimiao Zheng[1], Ming Gao[2] & Alexey L. Arkov[1]

[1]Department of Biological Sciences, Murray State University, Murray, KY, 42071, USA.
[2]Biology Department, Indiana University Northwest, Gary, IN, 46408, USA.

Correspondence and requests for materials should be addressed to Alexey L. Arkov (email: aarkov@murraystate.edu)

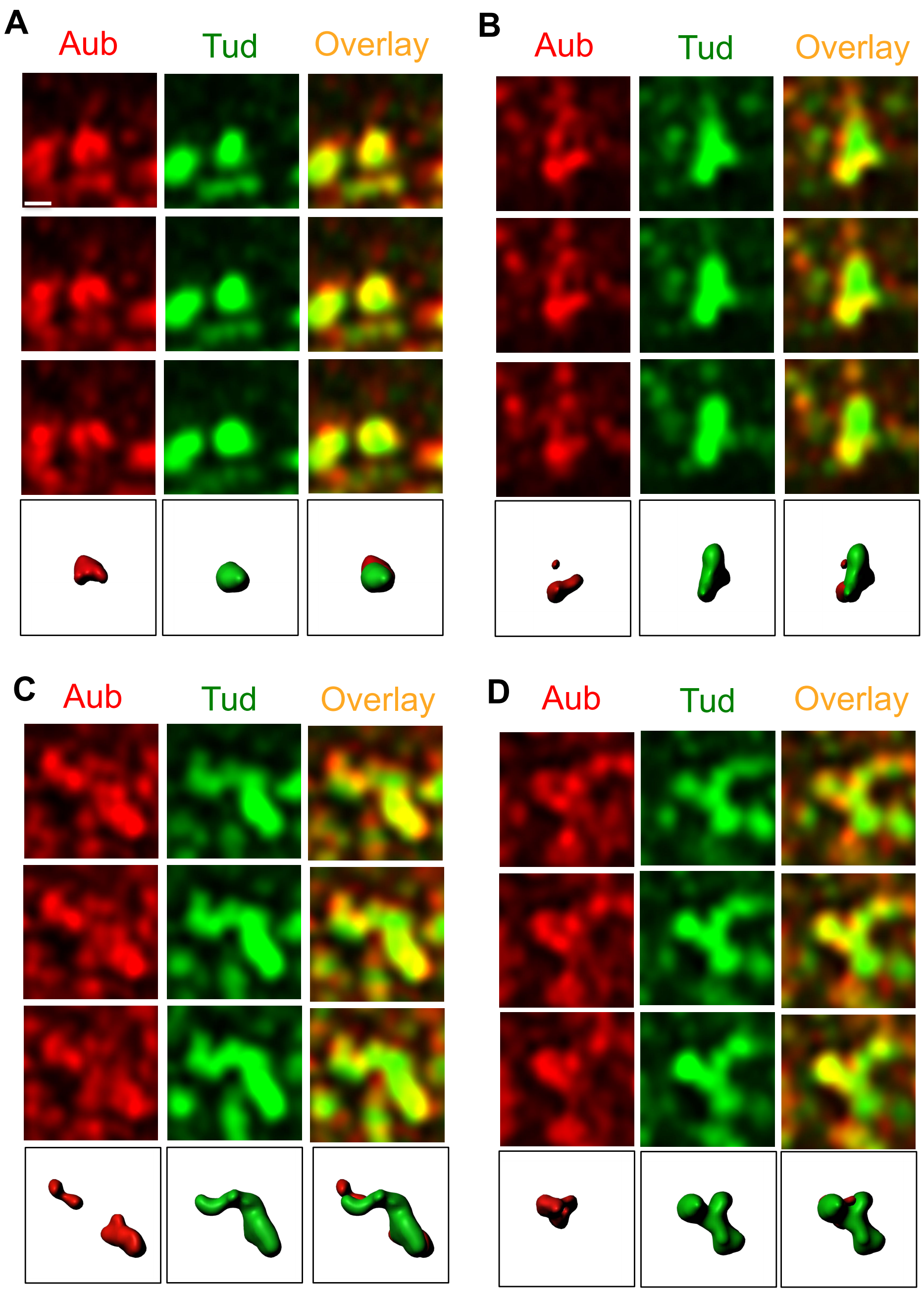

Supplementary Figure S1. A gallery of polar granules from the preblastoderm embryonic germ plasm. (**A-D**) Super-resolution confocal images of four different polar granules show distinct partially overlapping Aub (red channel) and Tud (green channel) clusters in different granules and their diverse morphology and size. Three consecutive optical sections are shown for each granule. Bottom panels show corresponding 3D reconstruction images. Scale bar in (A) is the same for all the panels in (**A-D**), including 3D reconstructions, and is 0.4 μm.

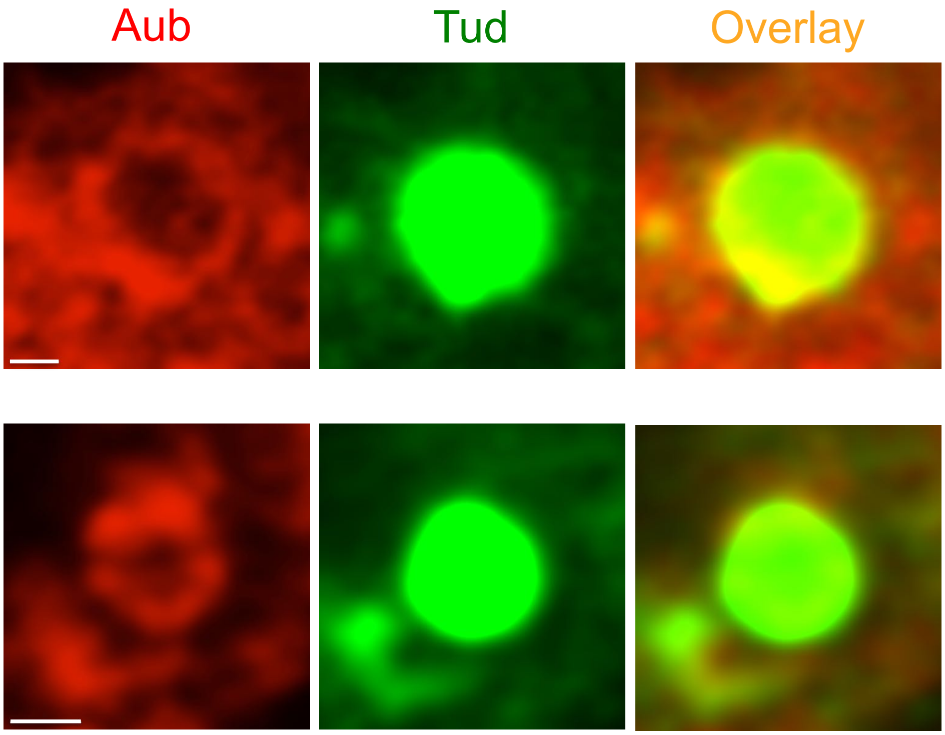

Supplementary Figure S2. Super-resolution confocal microscopy optical sections of different large cytoplasmic granules with Aub shell-Tud core architecture. Aub and Tud are imaged with red and green channels respectively. Scale bars shown at the Aub images for both granules are 0.5 μm.

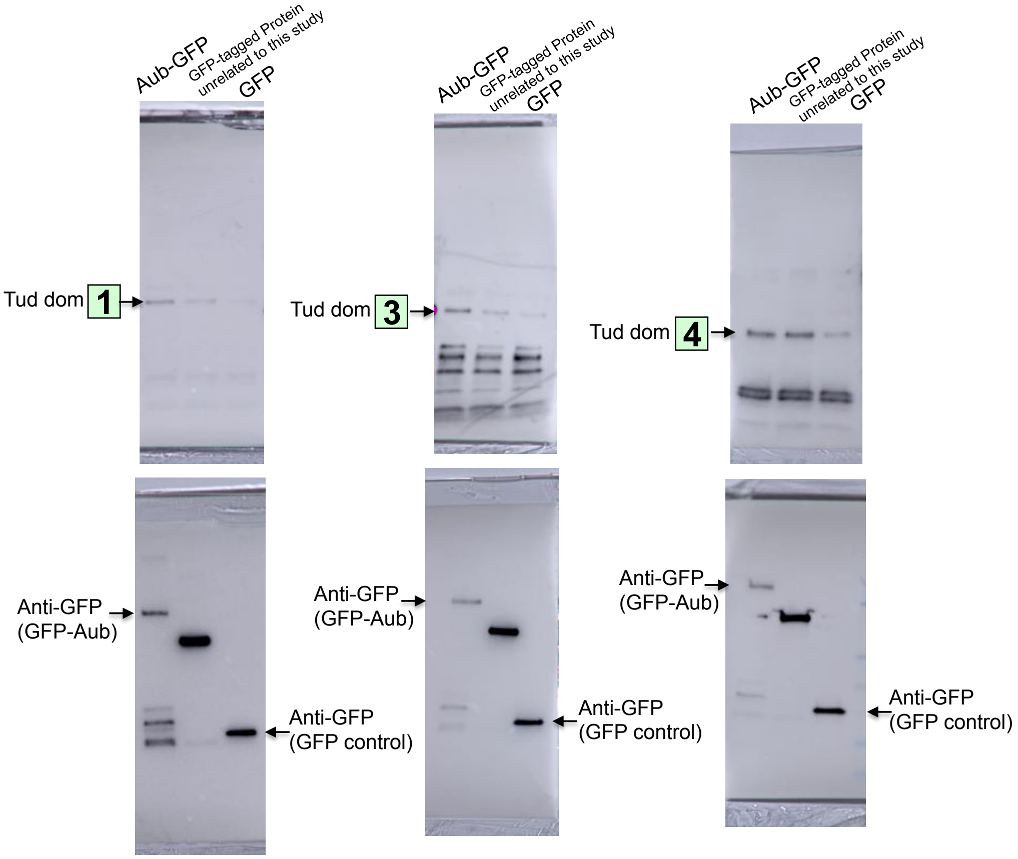

Supplementary Figure S3. Full-length western blots corresponding to the binding experiments for Tud domains 1,3 and 4 shown in Fig. 5B. A GFP-tagged protein in the middle lane was also tested for binding to Tud domains, however, it is not related to the current work.

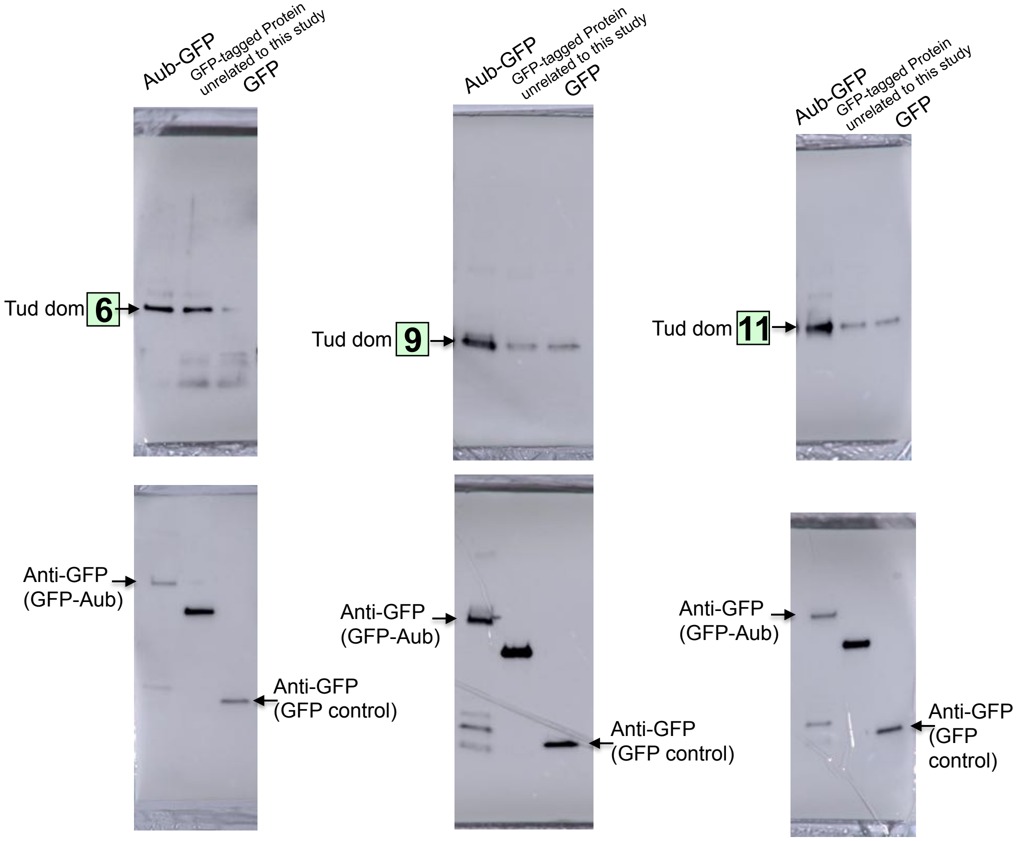

Supplementary Figure S4. Full-length western blots corresponding to the binding experiments for Tud domains 6, 9 and 11 shown in Fig. 5B. Similarly to Supplementary Fig. S3, an unrelated GFP-tagged protein in the middle lane was also tested for binding to Tud domains.

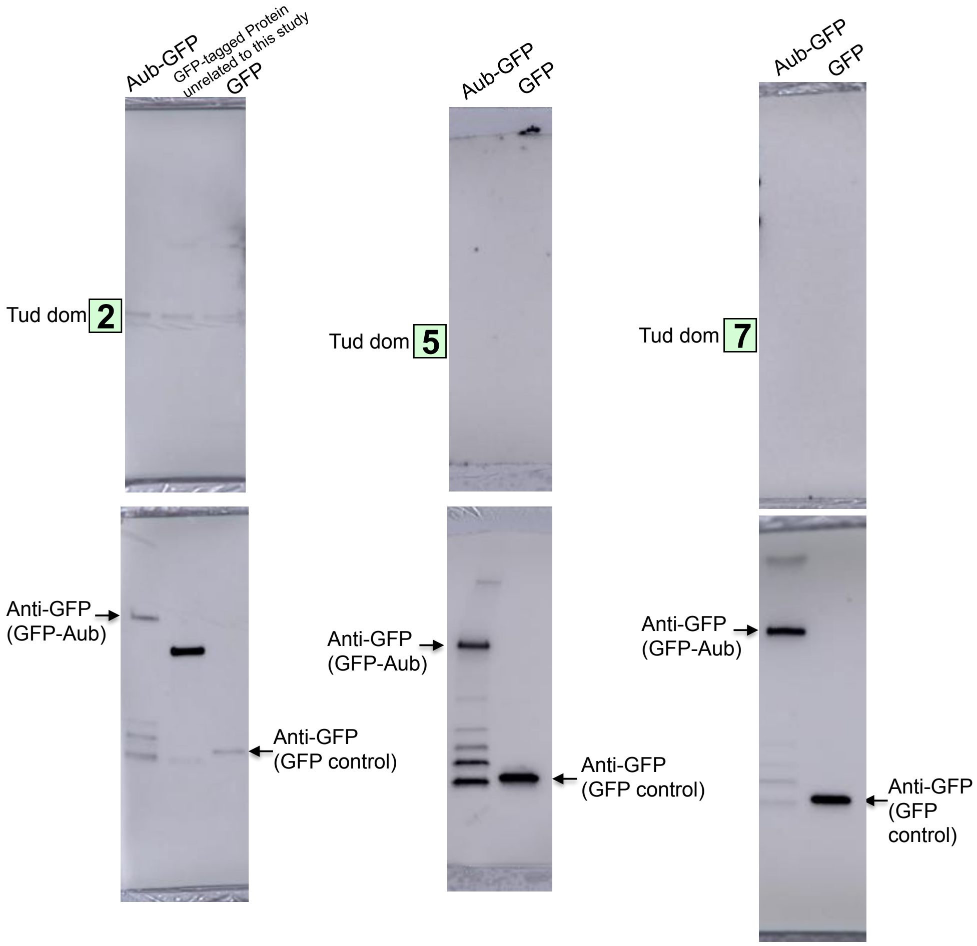

Supplementary Figure S5. Full-length western blots corresponding to the binding experiments for Tud domains 2, 5 and 7 that fail to show binding to Aubergine. Binding experiments and western blots were done as described for Fig. 5B. Top panels are western blots with anti-His antibody and bottom panels are western blots of the same gels probed with anti-GFP antibody to detect GFP-Aub and GFP control. A weak non-specific binding of Tud domain 2 to GFP-Aub and GFP control can be seen. An expected location for the other Tud domains is indicated on the left side of the anti-His antibody blots. Similarly to Supplementary Fig. S3 and Fig. S4, an unrelated GFP-tagged protein in the middle lane was also tested for binding to Tud domain 2.

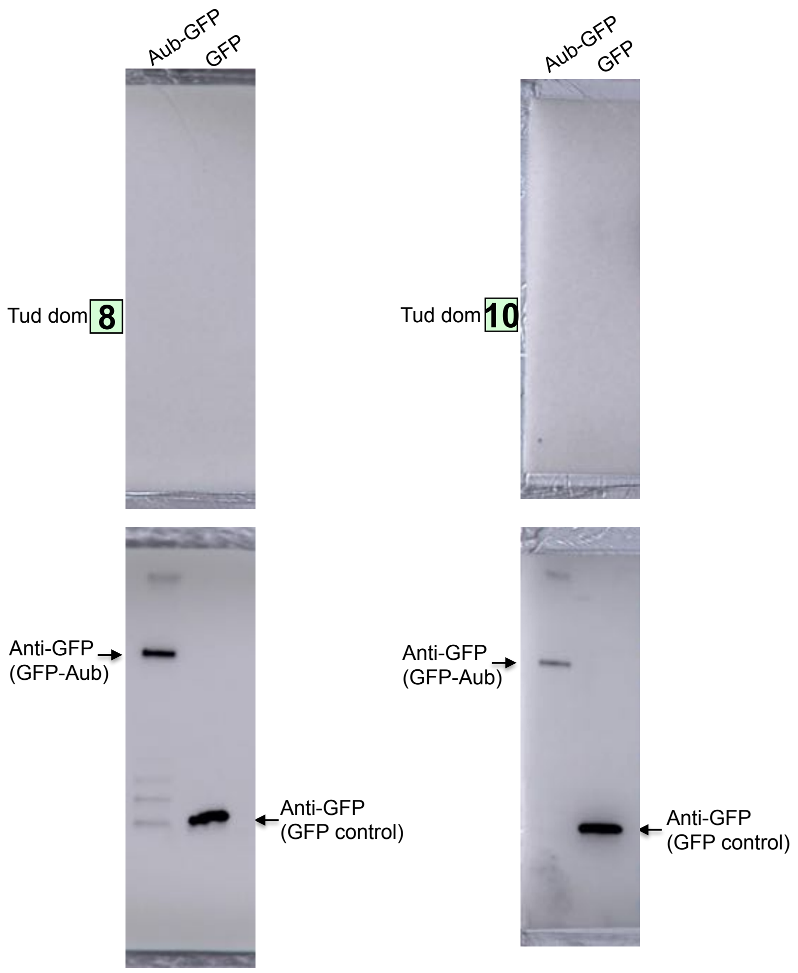

Supplementary Figure S6. Full-length western blots corresponding to the binding experiments for Tud domains 8 and 10 that fail to show binding to Aubergine. As in Fig. S5, top panels are anti-His antibody blots for a given Tud domain (expected locations of domains are indicated on the left) and bottom panels are anti-GFP antibody blots of the same gels which show GFP-Aub and GFP control used for binding reactions. All binding experiments were performed as described for Fig. 5B.
